# Sexual Health and Relationship Education Needs of People in Recovery for an Opioid Use Disorder

**DOI:** 10.1007/s11121-026-01909-z

**Published:** 2026-04-13

**Authors:** J. Michael Wilkerson, Kathryn R. Gallardo, I. Niles Zoschke, Hannah L. N. Stewart, Serena A. Rodriguez, Michael U. Anosike, Jason Pullin, Sheryl A. McCurdy

**Affiliations:** 1 Department of Health Promotion & Behavioral Sciences, The University of Texas Health Science Center (UTHealth) Houston School of Public Health, Austin, TX USA; 2 Department of Health Promotion & Behavioral Sciences, UTHealth Houston School of Public Health, Dallas, TX USA; 3 Department of Health Promotion & Behavioral Sciences, Center for Health Promotion & Prevention Research, UTHealth Houston School of Public Health, 7000 Fannin St., Suite 2620, Houston, TX 77030 USA; 4 Institute for Implementation Science, UTHealth Houston, Dallas, TX USA; 5 Alcohol Research Group, University of California at Berkley, Community Health Sciences Division, Emeryville, CA USA; 6 Recovery People, Harwood, TX USA

**Keywords:** Out-of-control sexual behavior, Hypersexuality, Drug use, Relapse, Recovery support services

## Abstract

People in recovery with histories of sex and drug-linked behavior (SDB) have an increased risk of substance use recurrence. However, sexual health concerns remain largely unaddressed by recovery support services researchers and practitioners. The purpose of this analysis was to describe the sex and relationship concerns of people in recovery for an opioid use disorder living in level II and level III recovery homes. Recovery homes are sober living homes that the National Association for Recovery Residences classifies into four levels based on staffing and service provision. We interviewed 93 residents and thematically analyzed 92 of the resulting transcripts; one was excluded because the participant did not talk about SDB. Most participants avoided sexual experiences or romantic relationships while living in recovery homes. Memories of SDB can trigger unwanted substance use recurrence, and histories of sexual trauma or reliance on drugs during sex impede connection with potential sex or romantic partners. Participants wanted to heal and prepare for healthy sexual or romantic relationships. Recovery residents could benefit from sexual health education that provides the skills for healthy sexual or romantic relationships.

## Background

People in early substance use recovery are advised to abstain from sexual or romantic relationships that might distract from focusing on recovery maintenance (Faulkner, 2004; Williams & Taleff, 2015). This advice has led to a social norm that discourages open conversations about sex and relationships among people in recovery. Fewer conversations are problematic because unaddressed sexual health concerns linked to previous substance use experiences can increase the risk of substance use recurrence (Ahuja et al., 2021; Boyle, 2023; Hall, 2019; Hallinan, 2020; Maehira et al., 2013; Ramos et al., 2021; Rawson et al., 2002; Tay et al., 2022).

Recovery support services, in general, and recovery homes, in particular, are potential settings for sexual health education programming. Mericle et al. (2019) reported that HIV services should be provided to men who have sex with men living in a recovery home. Recovery homes are sober living homes that offer four levels of services. The lowest level is less structured and self-governed, and the highest level is more structured and staffed with clinical personnel (National Association of Recovery Residences, 2019). Level II and III recovery homes create shared living environments informed by the social model of recovery (Borkman et al., 1998; Mericle et al., 2017, 2023; Polcin et al., 2023), have senior residents or house managers to oversee day-to-day operations, link residents to recovery support services within their communities, and offer various levels of life skills education (Substance Abuse and Mental Health Services Administration (SAMHSA), 2023). Level II and III homes that have life skills education are well-suited for a sexual health program.

Little has been written about how to support the sexual health of people in recovery, and to our knowledge, no research has focused on improving recovery outcomes among people with a history of SDB living in recovery homes. Most sex and drug-linked (SDB) programs have focused on HIV-related outcomes rather than substance use outcomes and have mostly targeted men who have sex with men in active use (Banbury et al., 2023; Choi et al., 2023; Herrijgers et al., 2024; Íncera-Fernández, 2025; Pozo-Herce et al., 2024). Further, most of these programs have been developed and implemented by clinicians in inpatient and outpatient environments.

Research indicates that relationship status and the substance use behaviors of romantic partners can positively or negatively influence a person’s substance use behavior (Leonard & Eiden, 2007; Muyingo et al., 2020). Alcohol research found that a romantic partner’s drinking was often predictive of one’s own alcohol use in both men and women (Mushquash et al., 2013). Relationship instability or dissolution also contributes to more frequent and heavier substance use (Smith et al., 2012; Whisman et al., 2022). These dynamics help explain why individuals in recovery demonstrate elevated risk of returning to active substance use when their partner also uses substances (Leonard & Eiden, 2007). Insights into partner influence led to calls for comprehensive substance use treatment programs that include romantic partners (Ariss & Fairbairn, 2020). Wiersma & Fischer, 2014).

Partner influence on substance use appears to differ by gender. Women appear to exert greater partner influences on their male partners, though they are also particularly susceptible to the risks associated with being in a relationship with discordant levels of substance use (Muyingo et al., 2020; Wiersma & Fischer, 2014). Women are less likely to be married when seeking substance use treatment services and often enter treatment with higher degrees of relationship volatility and less relational support than men (Tuchman, 2010). Once in recovery, men are at a higher risk of returning to substance use when their relationship status changes early in recovery (Permut et al., 2019). Factors that explain these differences include stressful life experiences (Keyes et al., 2011; Pilowsky et al., 2013), trauma (Collinson & Hall, 2021; Flasch et al., 2019), the strength of social networks (Collinson & Hall, 2021), and social support.

Braun-Harvey (2016a, 2016b) guides mental health providers wishing to integrate sexual health conversations into their clinical practice to work with clients to develop autonomous sexual pleasure. He describes autonomous sexual pleasure as the ability to healthfully experience sexual pleasure that aligns with one’s sexual values, free of self-loathing and judgment from others. Discussions about sexual autonomy necessitate that health education researchers and practitioners consider how autonomy is discussed within the human rights and sexual ethics literature. Within the human rights literature, sexual autonomy is bounded by a reasonable expectation of privacy in private and public settings (Valentiner, 2021). Within the field of sexual ethics, autonomy requires reciprocal bodily integrity, consent, trust, communication, and sexual pleasure in an environment free of social constraint (Marino, 2022; Reis et al., 2021). It is perhaps this latter component—freedom from social constraint—that is most difficult to achieve. It requires an understanding of how gender roles and societal norms constrain individual choice to engage in sexual and romantic relationships, and a striving to eliminate those ideas that constrain one or more partners’ choice of sexual expression and connection with others.

For sexual autonomy to be achieved, people need to be knowledgeable of sexual and relationship health information, have equitable access to medical and mental health care, and have the freedom to refuse or engage in sexual and relationship acts (Wittrock, 2023). A lack of sexual autonomy has been found to be associated with increased sexual distress (Alarcão, 2022). Braun-Harvey warns that people in recovery who lack sexual autonomy will rely upon maladaptive coping strategies when confronted with sexual situations that can trigger substance use recurrence. Braun-Harvey calls upon substance use treatment and recovery professionals to help people in treatment and recovery manifest autonomous sexual pleasure.

Faulkner (2004) recommends that people early in recovery wait at least two years before being sexually active or in a romantic relationship. However, she acknowledges that this advice is not realistic for many people. Faulkner’s book is one of the few resources written for a lay audience that offers sex and relationship advice for people in recovery. To better understand the sexual health education needs of people in early recovery and the desire for a life skills curriculum, in this paper, we describe the sex and relationship concerns of people living in level II and III recovery homes.

## Methods

### Parent Study

The parent study is an ongoing mixed-methods evaluation of 14 Level II and III MOUD-supportive recovery residences located in Austin, El Paso, Midland, San Angelo, and Houston, Texas, for people in recovery from an opioid use disorder. The study is being conducted by a Texas-based research team with international and domestic experience conducting research with individuals living with or in recovery from a substance use disorder, as well as substance use professionals. The qualitative evaluation employed ethnographic methods, including participant observation and interviews with residents and stakeholders. Information about the parent study protocol has been published elsewhere (Wilkerson et al., 2024a, 2024b), as have findings from other analyses of data from the parent study (Gallardo et al., 2024a, 2024b, 2025; Obekpa et al., 2023a, 2023b, 2024; Stewart et al., 2024, 2025; Wilkerson et al., 2024a, 2024b; Zoschke et al., 2025). In this analysis, we report results from in-depth interviews with residents. The University of Texas Health Science Center at Houston Committee for the Protection of Human Subjects (IRB) approved the research protocol and related study materials.

### Sampling and Recruitment

Between July 2021 and April 2023, a total of 316 people resided in one of the participating recovery homes. We conducted in-depth interviews with 92 residents with sexual experience. Eligible interview participants were currently or previously living in one of the participating homes, were 18 + years of age, and could complete the interview in English or Spanish; one participant completed the interview in Spanish in the presence of an interpreter who did live interpretation. Study staff contacted recovery residence operators and house managers, who then disseminated information about the study to their residents. Interested residents were then invited to schedule an interview by contacting the study team directly or scheduling through their house manager. Six team members—three investigators (JMW, KRG, SAM) and two graduate research assistants (INZ, HLNS), all with extensive training in qualitative data collection—conducted the interviews. Each interview began with a team member reviewing the consent form and obtaining verbal consent. Participation was voluntary. Residents were given a $25 gift card to compensate for their time.

### Data Collection

A semi-structured interview guide included questions about participants’ experiences living in the homes, taking medication for an opioid use disorder if relevant, and co-occurring health concerns, including the following questions about sexual health. Occasionally, the team revised the interview guide as new areas of inquiry emerged (Glaser & Strauss, 1999; Seidman, 2013). The portion of the interview that asked about sex and intimacy began with a question asking participants to tell the interviewer about how sex and drugs work together. Based on the participants’ responses, the interviewer probed for more information that explained how sex was different while high versus sober. We also asked how sex and intimate relationships had changed since becoming sober and how sex and intimate relationships got in the way or helped with recovery.

To minimize intrusions by non-participants, data were collected in person in a private area at the recovery residence. Approximately 23% (*N* = 21) of participants were interviewed virtually because of COVID-19 pandemic-related precautions or scheduling considerations. In these instances, participants joined a secure teleconferencing platform to participate in the interview. Interviewers conducted virtual interviews in private spaces, such as their on-campus office, using headphones as an additional privacy measure. Participants were encouraged to complete virtual interviews in a place where they had a degree of privacy, though they ultimately decided on the location from which they joined the interview. Interviews ranged from 23 to 97 min in length, with an average duration of 53 min; all were audio recorded with participants’ consent and transcribed verbatim. For the one participant who completed the interview with a Spanish-language interpreter, only the English portions were transcribed. Participants’ demographic data were collected using a brief demographic survey in a secure REDCap electronic data capture tool hosted at UTHealth Houston School of Public Health. The UTHealth Houston Committee for the Protection of Human Subjects, the university’s institutional review board, approved all study protocols.

### Analysis

Transcripts were uploaded into ATLAS.ti version 23 (ATLAS.ti Scientific Software Development GmbH, 2023) for analysis. Two evaluation team members developed preliminary structural and deductive codes (Saldaña, 2021) and then they independently applied the provisional codebook to three separate resident transcripts. The larger team met to compare how codes were applied and to add or modify codes to develop the codebook (Roberts et al., 2019). The two team members then re-coded data samples, using negotiated consensual validity to agree upon coding conventions (Sandelowski & Barroso, 2007). The first author then coded each interview. Weekly, the larger team met in peer debriefings (Nowell et al., 2017) to maintain rigor by discussing changes to the codebook and interview guide based on emerging patterns and draft analytic memos. From the coded transcripts, the first author generated ATLAS.ti reports for codes related to sexual health, e.g., *sexuality and romantic relationships in recovery*, *sexuality and romantic relationships in substance use*, and *sex and drugs*. The first author constructed preliminary themes and shared them with other evaluation team members to refine and finalize the thematic analysis (Tolley et al., 2016).

## Results

Data from 92 participants were included in the analysis (mean age = 37.5 years; standard deviation = 8.9 years). The majority were heterosexual (*n* = 80 (86.9%)) males (*n* = 55 (59.8%)). Most participants were Hispanic (*n* = 22 (23.9%)) or non-Hispanic whites (*n* = 62 (67.4%)). While most were single (*n* = 56 (60.9%)), 10 (10.9%) were married or living as married, one (1.1%) was in a committed relationship, 20 (21.7%) were divorced or separated, and three (3.3%) were widowed; two (2.2%) did not report their relationship status (Table 1).

**Table 1:** Sample characteristics of residents (*N* = 92)

	**Frequency**	**Percentage**
**Sex**		
Male	55	59.8
Female	34	36.9
Unknown/not reported	3	3.3
**Race/ethnicity**		
Non-Hispanic White	62	67.4
Hispanic	22	23.9
African American	1	1.1
Asian	1	1.1
Multi-racial	3	3.3
Unknown/not reported	3	3.3
**Sexual orientation**		
Straight/heterosexual	80	86.9
Gay/lesbian	4	4.3
Bisexual/pansexual	6	6.5
Unknown/not reported	2	2.2
**City**		
Austin	28	30.4
Houston	29	31.5
Midland	11	11.9
El Paso	18	19.6
San Angelo	6	6.5
**Marital status**		
Single	56	60.9
Married or living as married	10	10.9
Divorced or separated	20	21.7
Committed relationship	1	1.1
Widowed	3	3.3
Unknown/not reported	2	2.2
	**Mean**	**Standard deviation**
**Age, years**	37.5	8.9
**Length of time in recovery residence at interview, days**	108.6	93.8

Three themes described reasons people in recovery frequently avoid sexual experiences and romantic relationships. One theme described strategies some participants used or desired to use to heal and prepare for future healthy sexual experiences and romantic relationships.

### Why People in Recovery Avoid Sexual Experiences and Romantic Relationships

#### They Fear for Their Recovery

Participants were often advised by others in recovery to avoid sex and to not enter into a new relationship early in recovery to allow time to focus on healing themselves. Many of the participants we interviewed heeded this advice. One heterosexual woman explained what she believed were the potential risks of entering into relationships early in the recovery journey:

When people first enter recovery, they are looking for anything to fill that void [that substances used to occupy], and so, you know, sometimes that’s relationships, and they’ll jump in with a... with a man, or it’s sex, and they’ll just, you know… they’ll stay focused on that. And they’re not focused on themselves and their recovery.

Despite advice to remain single and sexually abstinent while in early recovery, some of the participants reported choosing to enter a new relationship. Other participants entered into recovery in an existing relationship.

The few participants in relationships described situations that sometimes benefited and sometimes harmed their recovery. For example, one heterosexual man described how he received support from his romantic partner when experiencing substance use cravings, “And then when there’s craves…when we [have] c…cravings or anything like that, we try to talk to each other and talk it out and stuff like that – encourage each other.” However, dating someone who struggles with a substance use disorder could jeopardize the recovery of both people in the relationship. For example, a heterosexual man told a story about meeting someone while he was in recovery who started using again. He attributed his romantic partner’s return to use to his own substance use recurrence: “She relapsed, and when she did, I ran, you know, ‘cause I didn’t wanna…I didn’t wanna get back on drugs. But within a couple weeks, I was drinking, and within a couple weeks, I was using.” This situation illustrated why many people in recovery have historically encouraged people new to recovery to avoid entering new relationships.

#### Fond Memories Can Trigger Recurrence

How participants described sex and substance use differed somewhat by substances used during sex and by gender. Most participants had polydrug use histories. Some participants with a history of stimulant use, especially methamphetamine use, reported enhanced sexual pleasure when having sex while using stimulants. Reports of experiencing sexual pleasure while using stimulants were more common among men than women. Many of the men who described these pleasurable experiences feared having sober sex because the desire for sex triggered substance use recurrence, as illustrated by the following quote from a bisexual/pansexual man:

Like, drinking will come into play with the sex. And then, you know, poppers all of a sudden are in the mix. And then before you know it, like, cocaine and meth make sense, and then, you know, you’re off to the races.

The same participant, when asked about the relationship between sex and drugs, talked about his fear of developing sex addiction or another addictive behavior if he did not first resolve the underlying issues that resulted in him first using drugs. He said, “It feels like there’s something missing in me, and if I don’t have drugs, then another addiction like sex is gonna try to jump in there.”

Some participants who found it pleasurable to have sex while using drugs expressed fear that any sexual behavior, even masturbation, might trigger pleasurable memories of having sex while on methamphetamine, resulting in cravings. In addition to these fears, some of the men we spoke with did not feel sex was or could be as pleasurable as the sex they had while using drugs. As illustrated by the following quote from a heterosexual man, some of the men felt more self-conscious when engaging in sex without the use of drugs:

It’s weird having sober sex… when you’re high, it’s just a different feeling, I guess…. Like, you’re not embarrassed to do certain things, you know what I mean? Like, you don’t give a crap when you’re high. You’ll do anything. Who cares what they think, you know? And when you’re sober, you’re kind of more… what’s the word, like held back, or more…. You know, like more… reserved.

Many participants stated that they missed the sex they had while uninhibited because they were less self-conscious and willing to engage in behaviors they might otherwise not do.

#### Loss of Sexual Autonomy Limits the Capacity for Intimacy

Stories about a loss of sexual autonomy due to previous sexual trauma were common among people of all genders and sexual orientations. Some of our participants described being abused as a child, while other participants described being abused as an adult while engaging in transactional sex or sex while using drugs. These participants frequently attributed their problematic sexual and substance use behaviors, including having a limited capacity for intimacy, to their traumatic experiences. For example, a heterosexual man said, “I was abused as a child, so I think it affected my sexuality. [It] made me very hypersexual …And when I got on amphetamines, that just enhanced everything.” Other participants described never having sober sex. One participant, for example, said, “I don’t think I’ve ever had sober sex” (heterosexual woman). This participant was unsure how to form connections with sex partners without the use of substances. While some participants attributed childhood or adult sexual abuse to feeling incapable of experiencing intimacy with a romantic partner, more participants, especially the women, described how having a history of transactional sexual encounters was a barrier to intimacy. For example, a bisexual/pansexual woman said, “While I’m sober, I’ve pretty much, like, avoided intimacy and sex.” While using drugs, she was coerced into engaging in sex work to repay her supplier for her drugs. As a result of the loss of agency she experienced from sexual coercion, she said, “It’s very hard for me to imagine that, like, I’m gonna fall in love with somebody, but, like, I guess you never know.” Similar statements were more common among women.

Among those with a history of exchanging sex for money or drugs, some expressed being unsure about sexual experiences in a romantic relationship where sex was not transactional. A quote from a heterosexual woman illustrates this feeling of uncertainty. In her interview, she explained how prior manipulative relationships made it hard to form healthy relationships and why she chose to abstain from sex or intimate relationships:

So, for the past three years, I’ve struggled with once I get into a relationship, battling that, “Oh, I think you should spend your money on me.” So, um, when I was working in 2018, I didn’t have that going on; I wasn’t trying to use men. But, like, right now, I’m in a hard situation. So, if I was to talk to a guy, [I would] automatically implement those old behaviors. So basically, sex and drugs, for me, is the same thing. Um, they’re both an addiction, and you can use both of them for manipulation and everything else. So, until I learn how to live correctly and do how I’m supposed to, I’m gonna stay abstinent from sex completely, and I don’t want drugs anymore, ever.

Nearly all of the participants we interviewed who told stories of experiencing sexual trauma or engaging in transactional sex to meet basic needs hesitated to have sex or be in an intimate relationship. For these participants, the loss of autonomy inhibited connection with romantic partners. Some of these participants communicated a desire to learn how to be in a healthy relationship.

### Strategies for Preparing for Future Healthy Relationships

While stories of accessing outside support to resolve problematic SDB were not common, two participants talked about participating in Sex and Love Addicts Anonymous (SLAA), a 12-step program for people living with sex addiction (see https://slaafws.org/):

I was really into SLAA. I had a sponsor for SLAA. She would put me on, like, 60-day restrictions from, like, calling men or anything like that, kinda just focusing on yourself and not worrying about a relationship. And, um, I still took heed to those tools that I learned upon this second go-round. (Heterosexual woman)

So, um, going to the SLAA meetings and, uh, having, like, these boundaries and rules, like vigorous honesty. The vigorous honesty part with the 12 steps, that, like, I see the benefits of that applying to my addiction to drugs and with sex and everything. Like, if it’s something that I can’t be honest about, then I’m not going to do it. (Bisexual/pansexual woman)

The quote from the first participant represented in this theme described a time in her past in which she participated in SLAA and how she is applying the skills she learned in her past to her current substance use recovery journey. Similarly, the quote from the second participant describes how her current participation in SLAA complements her substance use recovery.

Another participant described a ceremony her sponsor recommended she do to begin releasing pain associated with previous sexual encounters and to begin healing and opening up the possibility of a healthy relationship in the future:

We broke all soul ties I had to any men. So, um, you know, I took a tissue and tore it up for every man I’ve been with. And then when I was prostituting, we just did one…one tissue for that. And it felt…it was just like a relief for me. And I made a commitment, like, not to engage in any sexual activities again until I’m in a committed relationship. And, um…and, uh, you know, as long as I’m sober and in a committed relationship, and all my beliefs, and my standards, and my values, and all that are… It’s way different than… Like, I forgot I had all those, you know, ‘cause I’d been on drugs for so long. (Heterosexual woman)

While this was a unique experience described by only one participant, this quote illustrates the potential importance of ritual in marking a moment of transition. In this situation, the participant tore up the tissues—a proxy for her past life—and committed to a new value system, abstaining from sexual activities until in a committed relationship.

## Discussion

By describing the sex and relationship concerns of people living in level II and III recovery homes, we added to researchers’ and recovery support services professionals’ understanding of how to talk with people in recovery about sexual health training topics. When talking with residents about sexual health topics, it is important to understand that many are following advice they have been given to avoid sex or relationships when early in recovery, similar to the advice given by Faulkner (2004), so most conversations about sexual health topics should focus on planning for the future. However, for residents who are sexually active or in a relationship, immediate concerns about the impact on their recovery are salient (Ariss & Fairbairn, 2020; Leonard & Eiden, 2007; Mushquash et al., 2013; Muyingo et al., 2020; Smith et al., 2012; Torvik et al., 2013; Wiersma & Fischer, 2014), and these concerns can vary by gender (Permut et al., 2019; Tuchman, 2010; Walt et al., 2014). Prior research suggests, for example, that women may receive greater benefits from the relational support afforded by stable, supportive relationships, while also experiencing disproportionate risk of negative substance use outcomes following relationship dissolution (Leonard & Eiden, 2007; Smith et al., 2012). Men in stable relationships, on the other hand, may face higher risks of returning to use when their partner continues to use substances (Permut et al., 2019). Thus, researchers wanting to develop sexual health curricula for people in recovery or recovery support services professionals wanting to be better equipped to engage clients in sexual health conversations must be equipped to individually tailor curricula and dialogue.

Participants provided reasons why they avoided sex and relationships while early in recovery and expressed a desire to acquire the knowledge and skills needed to be in healthy sexual and romantic relationships in the future. Participants early in recovery were advised by people with more time in recovery to avoid sex and romantic relationships. However, without providing residents with new sexual health skills needed to resolve underlying sexual health concerns, it is plausible that, upon moving out of the recovery housing environment, some residents will experience preventable substance use recurrence when they once again become sexually active or romantically involved with other people. To avoid this outcome, recovery residents should be offered a life skills curriculum that imparts the knowledge and skills to prepare to be in healthy sexual or romantic relationships.

Some of our participants expressed a desire to develop new ways of thinking and acting, e.g., sexual scripts (Simon & Gagnon, 2003), that would allow them to experience sexual pleasure and form healthy relationships without the use of substances. However, many of our participants were unsure how to reconcile their previous sexual behavior with normative sexual scripts. Their uncertainty inhibited their ability to have the kinds of sex they enjoyed while using drugs; this was especially true for participants with a history of stimulant use. It is likely that some people in recovery no longer want to participate in some of the sexual behaviors they enjoyed while using drugs. People wishing to engage in new sexual behaviors likely need guidance on how to reevaluate their sexual values to consider which aspects of their value system are enhancing or inhibiting their ability to experience sexual pleasure, identify behaviors they find pleasurable while having sober sex, and communicate their sexual values and desires to potential sex partners. Educators providing this guidance must do so while being trauma-informed.

The need for trauma-informed care in providing mental health and substance use services is well documented (Bartholow & Huffman, 2023; Bendall et al., 2021; Han et al., 2021; Muskett, 2014). While there has been some attention to the need for trauma-informed sexual health curriculum, the majority of this work has focused on adolescent and young adult populations (Evans-Mitchell et al., 2025; Panisch et al., 2020) or on general counseling practices not specific to substance use (Fous et al., 2025). Early intervention development work in this space suggests that women in recovery may benefit from curriculum delivered in group settings and designed to facilitate peer-to-peer learning (Myers et al., 2018). Our findings suggest that while women and men may exhibit different sex and drug-linked behaviors, trauma is not bound to any one gender. We recommend additional intervention development work to better integrate best practices in trauma-informed care, sexual health, and substance use recovery. A trauma-informed curriculum should adhere to five principles: safety, collaboration, empowerment, trustworthiness, and choice (Bartholow & Huffman, 2023). The ideal curriculum would also be strengths-based and learner-centered, reducing power differential by placing the instructor in the role of facilitator rather than an expert and valuing the lived experiences of learners by integrating their stories into discussions and activities, teaching self-care, titrating/scaffolding exposure to new concepts, eliciting feedback, and creating social support outside the learning environment (Carello & Butler, 2015). A curriculum is needed that safely guides residents through the process of reflecting upon and often changing their sexual values, identifying new forms of sexual pleasure, and communicating their sexual values and desires to sexual and romantic partners.

Armed with knowledge and skills to have pleasurable sexual and romantic experiences without substances, people can then learn coping strategies to use when in situations that might otherwise trigger substance use recurrence. Learning and enacting new skills takes time and frequently requires interpersonal and environmental supports that reduce barriers and increase opportunities to practice and habituate to the newly learned skills (Conner & Norman, 2015; Glanz et al., 2015). If individuals fail to learn needed skills or find themselves in unsupportive environments, they are more likely to develop counterproductive, maladaptive coping responses to sustained recovery (Marlatt, 1996; Marlatt & George, 1984). Thus, a sexual health and recovery curriculum should be provided in a sex-positive environment in which people are comfortable speaking openly and building sexual autonomy (Braun-Harvey, 2016a, 2016b). Braun-Harvey (2011) calls upon substance use treatment and recovery professionals to employ five client-centered skills when working with people with a history of sexualized drug use: (1) suspend judgment and regulate emotional reactivity, (2) validate clients who fear engaging in sex could trigger substance use recurrence while affirming that everyone deserves to experience sexual pleasure, (3) remember that engaging in sex or being in a romantic relationship fulfills an essential human need so clients should be encouraged to explore what it means for them to have positive sexual and romantic experiences while sober, (4) understand how clients’ behaviors, such as going online to a triggering dating app, might be an attempt to address a sexual health concern, and that these behaviors can be an opportunity for the treatment and recovery professional to integrate a sex positive perspective into therapy, and (5) use motivational interviewing to support clients’ efforts to align their sexual and substance-using values so that any sexual or romantic behaviors support long-term recovery. Braun-Harvey’s recommendations were developed primarily to train treatment staff. However, his recommendations are likely transferable to recovery support services professionals who, if they learn to master these client-level skills, might be better equipped to talk with clients about SDB and improve recovery outcomes. Future research is needed to determine which of his recommendations are transferable to developing non-clinical life skills education for residents of a level II and level III recovery home.

### Study Limitations

This study has limitations. Data were collected from residents living in recovery homes across Texas. Qualitative research emphasizes transferability, or contextual relevance, rather than generalizability. Thus, researchers and practitioners deciding which of our findings are transferable to their context should be mindful of the demographic characteristics of the residents we interviewed and that the experience of living in a recovery home in Texas might be somewhat different from that in other states due to unaccounted-for cultural and environmental differences (Lincoln & Guba, 1985; Stalmeijer et al., 2024). Sexual health data were analyzed by one research team member (first author), who regularly met with coauthors throughout the analytic process for peer debriefings. While the discussions in these meetings reached consensus on the themes, a different research team would likely find similar and other meanings, as is typical of qualitative analysis. Despite these limitations, our findings are important, describing the sexual health needs of people living in recovery homes, which can inform the development of future sexual health education programs. Future research could extend these findings by replicating this study in their communities and by conducting quantitative studies that would allow for generalizability. Other limitations of our data were that few participants were in relationships or sought outside support to resolve problematic SDB. Future research could recruit a sample that includes more people in relationships and people who seek outside support to better understand how their sexual and relationship education needs differ from people who are single and do not seek outside support for SDB. Future research could also recruit a sample of people who would participate in a sexual health and relationship in recovery class and ask the participants what knowledge and skills they would want to learn by enrolling in the course.

## Conclusion

We recommend that recovery homes offer sexual health education as part of a life skills curriculum. To further reduce the frequency and severity of substance use recurrence events, recovery residents should be taught the skills they need to become sexually active and enter into romantic relationships without the use of substances whenever they are ready to do so. Data from some of our participants about informal sexual health conversations and healing rituals and comments from many of our participants about their reasons for avoiding sexual and romantic relationships indicate a desire for sexual health and recovery education. In collaboration with recovery home operators and staff, additional research is needed to develop culturally appropriate programs for recovery homes. Together, researchers and practitioners can provide recovery support services that are inclusive of non-judgmental conversations and skills training about sex and relationships for people in recovery.

## Data Availability

The data that support the findings of this study are part of an ongoing evaluation study. The data are not publicly available due to their containing information that could compromise the privacy of research participants. Data requests may be directed to the corresponding author (JMW).

## References

[CR1] Ahuja, N., Schmidt, M., Dillon, P. J., Alexander, A. C., & Kedia, S. (2021). Online narratives of methamphetamine use and risky sexual behavior: Can shame-free guilt aid in recovery? *Archives of Sexual Behavior,**50*(1), 323–332. 10.1007/s10508-020-01777-w 32671499 10.1007/s10508-020-01777-w

[CR2] Alarcão, V., Stefanovska-Petkovska, M., Candeias, P., & Pascoal, P. M. (2022). Exploring intersectional variations in sexual pleasure, sexual autonomy, and important correlates. *Social Sciences,**11*, Article 496. 10.3390/socsci11110496

[CR3] Ariss, T., & Fairbairn, C. E. (2020). The effect of significant other involvement in treatment for substance use disorders: A meta-analysis. *Journal of Consulting and Clinical Psychology,**88*(6), 526–540. 10.1037/ccp0000495 32162930 10.1037/ccp0000495 PMC7228856

[CR4] ATLAS.ti Scientifi c Software Development GmbH. (2023). *ATLAS.ti qualitative data analysis software (Version 25)*. https://atlasti.com

[CR5] Banbury, S., Lusher, J., Chandler, C., & Zervoulis, K. A. (2023). Pilot RCT of an online mindfulness-based cognitive intervention for chemsex. *Counselling and Psychotherapy Research,**24*(3), 994–1005. 10.1002/capr.12728

[CR6] Bartholow, L. A. M., & Huffman, R. T. (2023). The necessity of a trauma-informed paradigm in substance use disorder services. *Journal of the American Psychiatric Nurses Association,**29*(6), 470–476. 10.1177/10783903211036496 34334012 10.1177/10783903211036496

[CR7] Bendall, S., Eastwood, O., Cox, G., Farrelly-Rosch, A., Nicoll, H., Peters, W., Bailey, A. P., McGorry, P. D., & Scanlan, F. (2021). A systematic review and synthesis of trauma-informed care within outpatient and counseling health settings for young people. *Child Maltreatment,**26*(3), 313–324. 10.1177/1077559520927468 32536207 10.1177/1077559520927468

[CR8] Borkman, T. J., Kaskutas, A., Room, J., Bryan, K., & Barrows, D. (1998). An historical and developmental analysis of social model programs. *Journal of Substance Abuse Treatment,**15*(1), 7–17. 10.1016/S0740-5472(97)00244-4 9534122 10.1016/s0740-5472(97)00244-4

[CR9] Boyle, R. E. (2023). Like the first time, all over again”: Sex, relationships, and risk for relapse to substance use after release from prison. *Drugs Education, Prevention & Policy,**31*(4), 487–497. 10.1080/09687637.2023.2226811

[CR10] Braun-Harvey, D. (2011). *Sexual health in recovery: A professional counselor’s manual*. Springer Publishing Company.

[CR11] Braun-Harvey, D. (2016a). Sex outside the lines: Authentic sexuality in a sexually dysfunctional culture. *Journal of Sex & Marital Therapy,**42*(6), 566–567. 10.1080/0092623X.2016a.1176833 10.1080/0092623X.2016.1176833 27487048

[CR12] Braun-Harvey, D. (2016b). *Treating out of control sexual behavior: Rethinking sex addiction*. Springer Publishing Company. 10.1080/0092623X.2016.1210373 27487049

[CR13] Carello, J., & Butler, L. D. (2015). Practicing what we teach: Trauma-informed educational practice. *Journal of Teaching in Social Work,**35*(3), 262–278. 10.1080/08841233.2015.1030059

[CR14] Choi, E. P. H., Choi, K. W. Y., Wu, C., et al. (2023). Web-based harm reduction intervention for chemsex in men who have sex with men: Randomized controlled trial. *JMIR Public Health and Surveillance,**9*, Article e42902. 10.2196/42902 36602853 10.2196/42902 PMC9893729

[CR15] Collinson, B., & Hall, L. (2021). The role of social mechanisms of change in women’s addiction recovery trajectories. *Drugs Education, Prevention and Policy,**28*(5), 426–436. 10.1080/09687637.2021.1929077

[CR16] Conner, M., & Norman, P. (2015). *Predicting and changing health behaviour: Research and practice with social cognition models* (3rd ed.). Open University Press McGraw Hill Education.

[CR17] Evans-Mitchell, G. S., Sacca, L., Markham, C., Schultz, K., McCurdy, S., Tingey, L., & Peskin, M. F. (2025). A systematic review of trauma-informed sexual health education interventions for adolescents and young adults within the United States and Canada. *International Journal of Sexual Health,**37*(2), 198–208. 40400560 10.1080/19317611.2025.2464565 PMC12091932

[CR18] Faulkner, M. (2004). *Easy does it dating guide: For people in recovery*. Hazelden Publishing.

[CR19] Flasch, P., Boote, D., & Robinson, E. H. (2019). Considering and navigating new relationships during recovery from intimate partner violence. *Journal of Counseling and Development,**97*(2), 148–159. 10.1002/jcad.12246

[CR20] Fous, G., Bazzaroni, C., Needle, R., & Favasuli, S. (2025). Sexual integration therapy: A sex-positive sexual health model supporting authentic sexual expression, while addressing sexual compulsivity, shame, fear and trauma. *Sexual and Relationship Therapy,**40*(4), 747–772. 10.1080/14681994.2024.2359370

[CR21] Gallardo, K. R., Stewart, H. L. N., Pullin, J., Wilkerson, J. M., Neubauer, M. G., Kirzner, S. J., Thomas, I., Zoschke, I. N., Rodriguez, S. A., & McCurdy, S. A. (2025). Development and implementation of recovery housing policies and practices to support people taking medications for opioid use disorder. *Journal of Substance Use and Addiction Treatment*. 10.1016/j.josat.2025.209817 41125155 10.1016/j.josat.2025.209817

[CR22] Gallardo, K. R., Wilkerson, J. M., Stewart, H. L. N., Zoschke, I. N., Isaacs, C. F., & McCurdy, S. A. (2024a). “Being here is saving my life”: Resident experiences of living in recovery residences for people taking medication for an opioid use disorder. *Journal of Substance Use & Addiction Treatment,**158*, Article 209242. 10.1016/j.josat.2023.209242 38061632 10.1016/j.josat.2023.209242

[CR23] Gallardo, K. R., Zoschke, I. N., Stewart, H. L. N., Wilkerson, J. M., Henry, E. A., & McCurdy, S. A. (2024b). Supporting medication-assisted recovery in recovery residences: Staff support, managing built environment threats, and building a supportive network. *American Journal of Drug and Alcohol Abuse,**50*(5), 739–747. 10.1080/00952990.2024.2401983 39382549 10.1080/00952990.2024.2401983

[CR24] Glanz, K., Rimer, B. K., & Viswanath, K. (Eds.). (2015). *Health behavior and health education: Theory, research, and practice* (5th ed.). Jossey-Bass.

[CR25] Glaser, B. G., & Strauss, A. L. (1999). *Discovery of grounded theory: Strategies for qualitative research* (1st ed.). Taylor and Francis.

[CR26] Hall, P. (2019). *Understanding and treating sex and pornography addiction: A comprehensive guide for people who struggle with sex addiction and those who want to help them* (2nd ed.). Routledge. 10.4324/9781351112635

[CR27] Hallinan, R. (2020). Sexual function and alcohol and other drug use. In N. el-Guebaly, G. Carrà, M. Galanter, & A.M. Baldacchino (Eds.), *Textbook of addiction treatment* (pp. 1225–1239). Springer International Publishing. 10.1007/978-3-030-36391-8_85

[CR28] Han, H.-R., Miller, H. N., Nkimbeng, M., Budhathoki, C., Mikhael, T., Rivers, E., Gray, J. L., Trimble, K., Chow, S., & Wilson, P. (2021). Trauma informed interventions: A systematic review. *PLoS ONE,**16*(6), Article e0252747-e0252747. 10.1371/journal.pone.0252747 34157025 10.1371/journal.pone.0252747 PMC8219147

[CR29] Herrijgers, C., Verboon, P., Florence, E., Vandebosch, H., Poels, K., & Platteau, T. (2024). Assessing the effectiveness of an mHealth intervention to support men who have sex with men engaging in chemsex (Budd): Single-case and pre-post experimental design study. *JMIR Formative Research,**8*, Article e56606. 10.2196/56606 39365642 10.2196/56606 PMC11489797

[CR30] Íncera-Fernández, D., Riquelme, A. R., Sánchez-Ocaña, A., Montesinos, F., & Gámez-Guadix, M. (2025). A systematic review of intervention strategies aimed at chemsex users. *International Journal of Drug Policy,**140*, Article 104795. 10.1016/j.drugpo.2025.104795 40334305 10.1016/j.drugpo.2025.104795

[CR31] Keyes, K. M., Hatzenbuehler, M. L., & Hasin, D. S. (2011). Stressful life experiences, alcohol consumption, and alcohol use disorders: The epidemiologic evidence for four main types of stressors. *Psychopharmacology (Berl),**218*(1), 1–17. 10.1007/s00213-011-2236-1 21373787 10.1007/s00213-011-2236-1 PMC3755727

[CR32] Leonard, K. E., & Eiden, R. D. (2007). Marital and family processes in the context of alcohol use and alcohol disorders. *Annual Review of Clinical Psychology,**3*(1), 285–310. 10.1146/annurev.clinpsy.3.022806.091424 17716057 10.1146/annurev.clinpsy.3.022806.091424 PMC2667243

[CR33] Lincoln, Y. S., & Guba, E. G. (1985). *Naturalistic inquiry*. Sage Publications.

[CR34] Maehira, Y., Chowdhury, E. I., Reza, M., Drahozal, R., Gayen, T. K., Masud, I., Afrin, S., Takamura, N., & Azim, T. (2013). Factors associated with relapse into drug use among male and female attendees of a three-month drug detoxification-rehabilitation programme in Dhaka, Bangladesh: A prospective cohort study. *Harm Reduction Journal,**10*, Article 14. 10.1186/1477-7517-10-14 24004685 10.1186/1477-7517-10-14 PMC3846454

[CR35] Marino, P. (2022). Sexual use, sexual autonomy, and adaptive preferences: a social approach to sexual objectification. In Boonin, D. (Ed.), *The Palgrave Handbook of Sexual Ethics.* (pp. 111–128). Palgrave Macmillan. 10.1007/978-3-030-87786-6

[CR36] Marlatt, G. A. (1996). Models of relapse and relapse prevention: A commentary. *Experimental and Clinical Psychopharmacology,**4*(1), 55–60. 10.1037/1064-1297.4.1.55

[CR37] Marlatt, G. A., & George, W. H. (1984). Relapse prevention: Introduction and overview of the model. *Addiction,**79*(3), 261–273. 10.1111/j.1360-0443.1984.tb00274.x 10.1111/j.1360-0443.1984.tb00274.x 6595020

[CR38] Mericle, A. A., Carrico, A. W., Hemberg, J., Stall, R., & Polcin, D. L. (2019). Improving recovery outcomes among MSM: The potential role of recovery housing. *Journal of Substance Use,**24*(2), 140–146. 10.1080/14659891.2018.1523966 31213946 10.1080/14659891.2018.1523966 PMC6581465

[CR39] Mericle, A. A., Howell, J., Borkman, T., Subbaraman, M. S., Sanders, B. F., & Polcin, D. L. (2023). Social model recovery and recovery housing. *Addiction Research & Theory,**31*(5), 370–377. 10.1080/16066359.2023.2179996 37928886 10.1080/16066359.2023.2179996 PMC10624396

[CR40] Mericle, A. A., Polcin, D. L., Hemberg, J., & Miles, J. (2017). Recovery housing: Evolving models to address resident needs. *Journal of Psychoactive Drugs,**49*(4), 352–361. 10.1080/02791072.2017.1342154 28657823 10.1080/02791072.2017.1342154 PMC5998815

[CR41] Mushquash, A. R., Stewart, S. H., Sherry, S. B., Mackinnon, S. P., Antony, M. M., & Sherry, D. L. (2013). Heavy episodic drinking among dating partners: A longitudinal actor-partner interdependence model. *Psychology of Addictive Behaviors,**27*(1), 178–183. 10.1037/a0026653 22149955 10.1037/a0026653

[CR42] Muskett, C. (2014). Trauma-informed care in inpatient mental health settings: A review of the literature. *International Journal of Mental Health Nursing,**23*(1), 51–59. 10.1111/inm.12012 23363398 10.1111/inm.12012

[CR43] Muyingo, L., Smith, M. M., Sherry, S. B., McEachern, E., Leonard, K. E., & Stewart, S. H. (2020). Relationships on the rocks: A meta-analysis of romantic partner effects on alcohol use. *Psychology of Addictive Behaviors,**34*(6), 629–640. 10.1037/adb0000578 32271057 10.1037/adb0000578

[CR44] Myers, B., Carney, T., Browne, F. A., & Wechsberg, W. M. (2018). Development of a trauma-informed substance use and sexual risk reduction intervention for young South African women. *Patient Preference and Adherence,**12*, 1997–2006. 10.2147/PPA.S175852 30323569 10.2147/PPA.S175852 PMC6174905

[CR45] National Association of Recovery Residences. (2019). *NARR Standard 3.0 Compendium.* Retreived April 30, 2025 from https://narronline.org/wp-content/uploads/2019/02/NARR-Standard-Compendium-v3.pdf

[CR46] Nowell, L. S., Norris, J. M., White, D. E., & Moules, N. J. (2017). Thematic analysis: Striving to meet the trustworthiness criteria. *International Journal of Qualitative Methods,**16*(1), Article 10.1177/1609406917733847.

[CR47] Obekpa, E. O., McCurdy, S. A., Gallardo, K. R., Rodriguez, S. A., Ganduglia Cazaban, C., Brown, H. S., Yang, J. J., & Wilkerson, J. M. (2024). Characteristics and quality of life of people living with comorbid disorders in substance use recovery residences. *Frontiers in Public Health,**12*, Article 1412934. 10.3389/fpubh.2024.1412934 39628806 10.3389/fpubh.2024.1412934 PMC11611819

[CR48] Obekpa, E. O., McCurdy, S. A., Schick, V., Markham, C., Gallardo, K. R., & Wilkerson, J. M. (2023a). Situational confidence and recovery capital among recovery residents taking medications for opioid use disorder in Texas. *Journal of Addiction Medicine,**17*(6), 670–676. 10.1097/ADM.0000000000001206 37934528 10.1097/ADM.0000000000001206

[CR49] Obekpa, E. O., McCurdy, S. A., Schick, V., Markham, C., & Wilkerson, J. M. (2023b). Health-related quality of life and recovery capital among recovery residents taking medication for opioid use disorder in Texas. *Frontiers in Public Health,**11*, Article epub. 10.3389/fpubh.2023.1284192 10.3389/fpubh.2023.1284192 PMC10694473 38054070

[CR50] Panisch, L. S., Faulkner, M., Fernandez, S. B., & Fava, N. M. (2020). Exploring how trauma is addressed in sexual education interventions for youth: A scoping review. *Health Education & Behavior,**47*(6), 880–893. 32900237 10.1177/1090198120954398

[CR51] Permut, M., Greene, P., Stevens, E., & Jason, L. (2019). Gender differences in the association between romantic relationships and relapse among individuals in early recovery in Oxford House. *Alcoholism Treatment Quarterly,**37*(3), 302–314. 10.1080/07347324.2018.1544057

[CR52] Pilowsky, D. J., Keyes, K. M., Geier, T. J., Grant, B. F., & Hasin, D. S. (2013). Stressful life events and relapse among formerly alcohol dependent adults. *Social Work in Mental Health,**11*(2), 184–197. 10.1080/15332985.2012.711278 10.1080/15332985.2012.711278 PMC3808003 24167441

[CR53] Polcin, D. L., Mericle, A. A., Braucht, G. S., & Wittman, F. D. (2023). Moving social model recovery forward: Recent research on sober living houses. *Alcoholism Treatment Quarterly,**11*(2), 173–186. 10.1080/07347324.2023.2167528 10.1080/07347324.2023.2167528 PMC10139742 37125214

[CR54] Pozo-Herce, P. D., Martínez-Sabater, A., Sanchez-Palomares, P., et al. (2024). Effectiveness of harm reduction interventions in chemsex: A systematic review. *Healthcare (Basel)*. 10.3390/healthcare12141411 39057554 10.3390/healthcare12141411 PMC11275498

[CR55] Ramos, J. C., Azqueta, I., Soriano, M. T. H., Solano, J. F. C., & Ibarguchi, L. (2021). Sexual counselling and sexual therapy in chemsex users in an NGO in spain. *European Psychiatry,**64*(S1), Article S552-S552. 10.1192/j.eurpsy.2021.1472

[CR56] Rawson, R. A., Washton, A., Domier, C. P., & Reiber, C. (2002). Drugs and sexual effects: Role of drug type and gender. *Journal of Substance Abuse Treatment,**22*(2), 103–108. 10.1016/s0740-5472(01)00215-x 11932136 10.1016/s0740-5472(01)00215-x

[CR57] Reis, J., de Oliveira, L., Oliveira, C., & Nobre, P. (2021). Psychosocial and behavioral aspects of women’s sexual pleasure: A scoping review. *International Journal of Sexual Health,**33*(4), 494–515. 10.1080/19317611.2021.1910890 38595786 10.1080/19317611.2021.1910890 PMC10903595

[CR58] Roberts, K., Dowell, A., & Nie, J.-B. (2019). Attempting rigour and replicability in thematic analysis of qualitative research data: A case study of codebook development. *BMC Medical Research Methodology,**19*(1), Article 66. 10.1186/s12874-019-0707-y 30922220 10.1186/s12874-019-0707-y PMC6437927

[CR59] Saldaña, J. (2021). *The coding manual for qualitative researchers*. Sage.

[CR60] Sandelowski, M., & Barroso, J. (2007). *Handbook for synthesizing qualitative research*. Springer.

[CR61] Seidman, I. (2013). *Interviewing as qualitative research: A guide for researchers in education and the social sciences* (4th ed.). Teachers College Press.

[CR62] Simon, W., & Gagnon, J. H. (2003). Sexual scripts: Origins, influences and changes. *Qualitative Sociology,**26*(4), 491–497. 10.1023/B:QUAS.0000005053.99846.e5

[CR63] Smith, P. H., Homish, G. G., Leonard, K. E., & Cornelius, J. R. (2012). Women ending marriage to a problem drinking partner decrease their own risk for problem drinking. *Addiction,**107*(8), 1453–1461. 10.1111/j.1360-0443.2012.03840.x 22321045 10.1111/j.1360-0443.2012.03840.x

[CR64] Stalmeijer, R. E., Brown, M. E., & O’Brien, B. C. (2024). How to discuss transferability of qualitative research in health professions education. *The Clinical Teacher,**21*(6), Article e13762. 10.1111/tct.13762 38497107 10.1111/tct.13762

[CR65] Stewart, H.L.N., Gallardo, K.R., Niedenfuehr, J.M., Zoschke, I.N., Wilkerson, J.M., Gillespie, D., Kaduri, P., Rodriguez, S.A., & McCurdy, S.A. (2025). The intersection of medications for opioid use disorder and mental health in substance use recovery housing in a U.S. setting. *Journal of Drug Issues*. 10.1177/00220426251346673

[CR66] Stewart, H. L. N., Wilkerson, J. M., Gallardo, K. R., Zoschke, I. N., Gillespie, D., Rodriguez, S. A., & McCurdy, S. A. (2024). “And now that i feel safe…i’m coming out of fight or flight”: A qualitative exploration of challenges and opportunities for residents’ mental health in substance use recovery housing. *Community Mental Health Journal,**60*(8), 1484–1492. 10.1007/s10597-024-01301-7 38822922 10.1007/s10597-024-01301-7

[CR67] Substance Abuse and Mental Health Services Administration (SAMHSA). (2023). *Best practices for recovery housing*. (PEP23–10–00–002). Rockville, MD

[CR68] Tay, E., Lo, W. K. W., & Murnion, B. (2022). Current insights on the impact of gamma-hydroxybutyrate (GHB) abuse. *Substance Abuse and Rehabilitation,**13*, 13–23. 10.2147/sar.S315720 35173515 10.2147/SAR.S315720 PMC8843350

[CR69] Tolley, E. E., Ulin, P. R., Mack, N., Robinson, E. T., & Succop, S. M. (2016). *Qualitative methods in public health: A field guide for applied research*. John Wiley & Sons.

[CR70] Torvik, F. A., Røysamb, E., Gustavson, K., Idstad, M., & Tambs, K. (2013). Discordant and concordant alcohol use in spouses as predictors of marital dissolution in the general population: Results from the Hunt Study. *Alcoholism, Clinical and Experimental Research,**37*(5), 877–884. 10.1111/acer.12029 23384147 10.1111/acer.12029

[CR71] Tuchman, E. (2010). Women and addiction: The importance of gender issues in substance abuse research. *Journal of Addictive Diseases,**29*(2), 127–138. 10.1080/10550881003684582 20407972 10.1080/10550881003684582

[CR72] Valentiner, D. (2021). The human right to sexual autonomy. *German Law Journal,**22*, 703–717. 10.1017/glj.2021.35

[CR73] Whisman, M. A., Salinger, J. M., & Sbarra, D. A. (2022). Relationship dissolution and psychopathology. *Current Opinion in Psychology,**43*, 199–204. 10.1016/j.copsyc.2021.07.016 34416683 10.1016/j.copsyc.2021.07.016

[CR74] Wiersma, J. D., & Fischer, J. L. (2014). Young adult drinking partnerships: Alcohol-related consequences and relationship problems six years later. *Journal of Studies on Alcohol and Drugs,**75*(4), 704–712. 10.15288/jsad.2014.75.704 24988269 10.15288/jsad.2014.75.704 PMC4108609

[CR75] Wilkerson, J. M., Atkinson, J., Akkala, S., Zoschke, I. N., Anosike, M. U., Gallardo, K. R., Rodriguez, S. A., Brown, H. S., Ganduglia Cazaban, C., Yang, J., Herrera, E., Howell, J., & McCurdy, S. A. (2024). Recovery residences are an innovative site for HIV prevention interventions targeting people who inject drugs: Preliminary data from Project HOMES. *AIDS Education and Prevention*. 10.1521/aeap.2024.36.6.403 39705177 10.1521/aeap.2024.36.6.403

[CR76] Wilkerson, J. M., Gallardo, K. R., Rodriguez, S. A., Brown, H. S., Ganduglia Cazaban, C., Yang, J. J., Herrera, E. R., Zoschke, I. N., Stewart, H. L. N., & McCurdy, S. A. (2024). Expansion and evaluation of level II and III recovery residences for people taking medications for an opioid use disorder: Project HOMES (Housing for MAR Expanded Services) study protocol. *BMJ Open*. 10.1136/bmjopen-2024-084115 39496371 10.1136/bmjopen-2024-084115 PMC11535685

[CR77] Williams, I. L., & Taleff, M. J. (2015). Sex, romance, and dating in treatment recovery: Ethical reflections and clinical deliberations on challenging addiction decision making. *Journal of Ethics in Mental Health,**9*, 1–7.

[CR78] Wittrock, J. (2024). A human right to pleasure? Sexuality, autonomy and egalitarian strategies. *Journal of Medical Ethics,**50*, 263–267. 10.1136/jme-2023-109011 37277174 10.1136/jme-2023-109011 PMC10958316

[CR79] Zoschke, I. N., Gallardo, K. R., Gillespie, D., Stewart, H. L. N., Rodriguez, S. A., McCurdy, S. A., & Wilkerson, J. M. (2025). Sustaining substance use recovery housing for people taking medications for opioid use disorder: Diverse funding, strategic partnerships, and charging rent. *Frontiers in Public Health,**13*(30), Article 1528971. 10.3389/fpubh.2025.1528971 40371296 10.3389/fpubh.2025.1528971 PMC12075534

